# Anaesthesiologic protocol for kidney transplantation in two patients with Fabry Disease: a case series

**DOI:** 10.1186/1757-1626-1-321

**Published:** 2008-11-18

**Authors:** Massimiliano Sorbello, Massimiliano Veroux, Melania Cutuli, Gianluigi Morello, Annalaura Paratore, Mirko Tindaro Sidoti, Jessica Giuseppina Maugeri, Massimiliano Gagliano, Giuseppe Giuffrida, Daniela Corona, Pierfrancesco Veroux

**Affiliations:** 1 Department of Surgery, Transplantation and Advanced Technologies, Anaesthesia and Intensive Care Unit, University Hospital of Catania, Via Santa Sofia, 86 95123 Catania, Italy; 2 Department of Surgery, Transplantation and Advanced Technologies, Vascular Surgery and Organ Transplant Unit, University Hospital of Catania, Via Santa Sofia, 86 95123 Catania, Italy

## Abstract

Fabry's Disease is a rare genetic syndrome, with a classic X-linked alpha -galactosidase A deficiency phenotype, responsible for glico-sphyngolypids metabolism impairment with clinical effects in several organs and functions. We describe the anaesthesiologic implications of two patients with Fabry disease who underwent a kidney transplantation from a deceased donor. We recommend careful preoperative evaluation, including cardiac sonography study and spirometry for Fabry disease patients, and according to our experience, we recommend advanced haemodynamic monitoring during surgery. Careful airway examination should be further performed, with particular attention to patient ventilability prediction and available alternative strategies for airway management in case of difficulties. A nephroprotective strategy and a particular care to the associated end-stage organ disease may significantly improve the long-term outcome of patients with Fabry Disease.

## Introduction

Fabry Disease (FD) is a rare genetic syndrome, with a classic X-linked alpha -galactosidase A (alpha -Gal A) deficiency phenotype; it has an estimated incidence of approximately 1 in 40,000 – 50,000 males, with recent epidemiologic evidence of later-onset variants [1]. The underlying enzymatic defect consists in lisosomial alpha galactosidase deficit, responsible for glico-sphyngolypids metabolism impairment resulting in the accumulation of uncleaved glycosphingolipids in many human cell types, with clinical effects in several organs and functions. The enzymatic defect leads to progressive accumulation of glycosphingolipids in vascular endothelial cells, renal cells and cardiomyocytes. Classically, homozygous young males are severely affected by Fabry disease, whose manifestations appear during early years of extra-uterine life.

Proteinuria usually manifests before the fourth decade of life and rapidly progresses to end-stage renal disease requiring dialysis and kidney transplantation [1]. Kidney transplantation is the best available method to restore a normal renal function with an excellent long term graft and patient survival, and it improves extrarenal clinical symptoms of Fabry disease [2,3]. Patients with Fabry disease are at high surgical risk, due to impaired renal and respiratory function, cerebrovascular and cardiovascular disease, and intraoperative management may be challenging. However, very little is known about anaesthetic implication in the management of patients with Fabry disease who underwent a kidney transplantation [4,5]

## Case presentations

We herein describe two patients with Fabry disease who underwent a deceased donor kidney transplantation, by focusing on the anaesthesiologic implications for this rare disease.

### Case 1

42 year-old male patient, ASA III; positive anamnesis for hypertension, left ventricular hypertrophy, incomplete right bundle block, moderate mitralic and aortic insufficiency; end-stage kidney disease in dialysis for 5 years. Diffuse angiokeratomas, especially in lower limbs and inferior abdomen.

### Case 2

53 years old male patient, ASA III; myocardial infarction 3 years before, no revascularization or angioplasty. Hypertension, chronic obstructive lung disease with borderline spirometry and end-stage renal disease in dialysis for 8 years. Diffuse angiokeratomas in lower limbs, abdomen and thorax.

Anaesthesiological implications for both patients might be relevant: however, despite the underlying Fabry disease, standard anaesthesiological protocol for kidney transplantation was applied: propofol 1.5 mg*kg^-1^, cisatracurium 2 mg*kg^-1 ^and fentanil 1.5 mcg*kg^-1 ^were administered for anaesthesia induction, while maintenance was performed with sevoflurane 1–1.5 MAC and fentanil/cisatracurium boluses on demand. Accordingly to our protocol, fenoldopam continuous infusion was performed up to 3rd postoperative day; one of two patients received "nephroprotective" infusion at standard 0.1 mcg*kg^-1 ^*min^-1^, while one patient received scalar doses up to 0.8 mcg*kg^-1 ^*min^-1 ^to control unresponsive hypertension. Cardio-haemodinamic management was standard 3 leads EKG, invasive blood pressure (difficult arterial cannulation in both cases) and in the second patient it included non invasive cardiac output measurement via PRAM^® ^monitor (arterial waveform advanced analysis). Careful haemodynamic monitoring was useful for both intra-operative fluid management and to titrate anti-hypertensive therapy. Both patients received intraoperative transdermal nitroglycerine during surgery.

Interestingly, one patient showed unexpected laryngoscopic and intubation difficulty, despite normal antropometric predictive parameters. Ventilation was always granted and intubation was achieved at third attempt using McCoy blade and Frova Hollow introducer (Cook^® ^– Bloomington – USA).

In both patients awakening was uneventful after uncomplicated surgery; extubation was performed in operative room and incentival spirometry was started immediately the day after surgery. Analgesia was granted with intravenous morphine.

In both cases renal function restored on day 2 and 3 post-transplant, respectively, with no needs for dialysis. At a follow up of 52 months and 13 months, respectively, both recipients are alive, in treatment with algasidase beta replacement therapy, and have an excellent renal function.

## Discussion

Renal pathology is one of the hallmarks of FD and is the most frequent cause of death, usually when patients are aged 30–50 years. Polyuria due to concentration defects can be among the first manifestations of kidney involvement, and it is always accompanied or immediately followed by rapid evolving proteinuria [1].

Global concentric ventricular hypertrophy, valvular abnormalities and cardiac conduction disturbance are common in affected males older than 30 years [6]. Moreover, disease of the airways appears to be significantly worse in male patients and smokers[7].

In conclusion, preoperative assessment of FD should concentrate on end-stage organ damage to the hearth, brain, lungs and kidneys. Patients with Fabry disease who are undergoing a kidney transplantation need a careful preoperative evaluation of cardio-pulmonary functionality, including echo-cardiography and spirometry, and an advanced haemodynamic monitoring during surgery, to prevent severe cardiovascular and respiratory impairment. Our clinical reports demonstrated that a standard anaesthesiological protocol could be applied in these patients. However, a special care should be addressed to the treatment of concomitant pathologies, which may significantly adverse the early postoperative outcome. A careful airway examination should be further performed, with particular attention to patient ventilability prediction and available alternative strategies for airway management in difficult cases. Finally, a nephroprotective strategy should be applied to all recipients to improve the long term outcome of transplanted patients with Fabry disease.

## Abbreviations

ASA: American Society of Anaesthesiology; MAC: Minimum alveolar concentration; FD: Fabry disease

## Consent

Written informed consent was obtained from the patients for publication of this case report. A copy of the written consent is available for review by The Editor-in-chief of this journal.

## Competing interests

The authors declare that they have no competing interests.

## Authors' contributions

MS performed the anaesthesiologic protocol, was a major contributor in writing the manuscript and has given the final approval to the manuscript. MV performed the kidney transplantation, was a major contributor in writing the manuscript and has given the final approval to the manuscript. MC performed the anaesthesiologic protocol and analyzed and interpreted the data regarding the post-transplant course. GM performed the anaesthesiologic protocol and analyzed and interpreted the data regarding the post-transplant course. AP interpreted the intraoperative parameters during the transplant procedure. MTS was responsible of the postoperative management of the transplant recipients. JGM interpreted and adapted the enzyme replacement therapy in transplant patients. MG interpreted the data regarding the follow up of the transplant recipient. GG interpreted the data regarding the follow up of the transplant recipient; DC: interpreted the laboratory data. PV performed the kidney transplantation, was a major contributor in writing the manuscript and has given the final approval to the manuscript. All the authors read and approved the final manuscript
